# Thin Air Resulting in High Pressure: Mountain Sickness and Hypoxia-Induced Pulmonary Hypertension

**DOI:** 10.1155/2017/8381653

**Published:** 2017-03-27

**Authors:** Jan Grimminger, Manuel Richter, Khodr Tello, Natascha Sommer, Henning Gall, Hossein Ardeschir Ghofrani

**Affiliations:** ^1^Department of Internal Medicine, University Clinic Hamburg Eppendorf, University of Hamburg, Martinistrasse 52, 20246 Hamburg, Germany; ^2^Department of Internal Medicine, Justus Liebig University Giessen, Universities of Giessen and Marburg Lung Center (UGMLC), Klinikstrasse 33, 35392 Giessen, Germany; ^3^German Center for Lung Research (DZL), Giessen, Germany; ^4^Department of Pneumology, Kerckhoff Heart and Thoracic Center, Bad Nauheim, Germany; ^5^Department of Medicine, Imperial College London, London, UK

## Abstract

With rising altitude the partial pressure of oxygen falls. This phenomenon leads to hypobaric hypoxia at high altitude. Since more than 140 million people permanently live at heights above 2500 m and more than 35 million travel to these heights each year, understanding the mechanisms resulting in acute or chronic maladaptation of the human body to these circumstances is crucial. This review summarizes current knowledge of the body's acute response to these circumstances, possible complications and their treatment, and health care issues resulting from long-term exposure to high altitude. It furthermore describes the characteristic mechanisms of adaptation to life in hypobaric hypoxia expressed by the three major ethnic groups permanently dwelling at high altitude. We additionally summarize current knowledge regarding possible treatment options for hypoxia-induced pulmonary hypertension by reviewing in vitro, rodent, and human studies in this area of research.

## 1. The Importance of High Altitude Medicine

With rising altitude, atmospheric pressure falls. The percentage of oxygen in the air (20.9%) is mostly independent of region and height [1], but since gases (in contrast to liquids) are compressible, the partial pressure of oxygen (PO_2_) falls with rising altitude, resulting in hypobaric hypoxia at high altitude [2, 3]. PO_2_ at sea level is approximately 159 mm Hg, whereas on the peak of Mount Everest PO_2_ is only about 53 mm Hg [4, 5]. Acute exposure to hypoxic conditions, dependent on the severity, may lead to acute mountain sickness (AMS) and even life-threatening conditions such as high altitude cerebral edema (HACE, which can occur as the end-stage of AMS) and high altitude pulmonary edema (HAPE) [6, 7]. Long-term exposure to hypobaric hypoxia may also lead to health problems in the form of chronic mountain sickness (CMS) [8]. Pulmonary hypertension (PH) occurs in several altitude-associated diseases including CMS and is a key feature of HAPE [9–11]. AMS as well as CMS occur at heights above 2500 m [6–8, 12], and altitude is categorized based on such physiological changes (Table 1) [13].

With approximately 35 million people traveling to high altitude each year, as well as military and rescue personnel, the latter often without adequate time to acclimatize, knowledge of the acute forms of maladaptation to hypoxia as well as their treatment is important [14]. CMS as the chronic form of maladaptation to high altitude conditions is also not to be neglected as a healthcare problem, because there are currently more than 140 million people permanently living at altitudes above 2500 m [15].

## 2. Acute Reactions of the Cardiopulmonary System to Hypoxia

### 2.1. Physiologic Response

Within the first seconds after exposure to hypoxia, the resting cardiac output (CO) is increased. In 1982, Naeije et al. [26] demonstrated that this phenomenon was entirely based on the rise of heart rate and that stroke volume remained unchanged [26]. Furthermore, the increase in CO matched the decrease in arterial oxygen concentration. This phenomenon only lasts for a few days. CO then returns almost to sea-level values, with heart rate remaining elevated and stroke volume decreasing [27, 28]. One reason for this adaptation is an increase in sympathetic nervous system activity along with decreased parasympathetic activity [29, 30]. An additional response to hypoxia is a rise in breathing frequency as well as tidal volume, also known as the hypoxic ventilatory response (HVR). This mechanism can increase alveolar ventilation by as much as 5-fold. HVR is triggered through chemoreceptors located in the bifurcation of the carotid arteries and is a direct response to the decreased partial pressure of arterial oxygen [4, 31]. Resting ventilation returns to low-altitude levels after several days [32].

Within the first hours of exposure to hypobaric hypoxia at high altitude, erythrocyte concentration increases because of a reduction in plasma volume due to dehydration [31]. The latter is caused by tachypnea in dry high-altitude air [33] and by increased diuresis [34, 35]. After this immediate response, circulating erythropoietin levels become vastly elevated within the first 24–48 hours of exposure to hypoxia and return to baseline values by the end of the first week [36]. The resulting elevation in erythrocyte concentration can be seen after 3-4 weeks [37].

In order for all these compensatory mechanisms to work adequately, acclimatization to high altitude is necessary. It is therefore recommended that people traveling above 3000 m ascend only about 300–500 m per day with a day of rest every third to fourth day [7].

### 2.2. Hypoxic Pulmonary Vasoconstriction: Clinical Significance and Molecular Mechanisms

Acute and prolonged exposure to hypoxia, for example, during a stay at high altitude, results in increased pulmonary vascular resistance and increased afterload of the right ventricle due to a mechanism termed hypoxic pulmonary vasoconstriction (HPV), also known as the von-Euler-Liljestrand mechanism. HPV is induced below a PO_2_ of about 100 mm Hg (depending on species) and has two phases: an initial pulmonary vasoconstriction occurs within seconds after the onset of hypoxia, peaks after several minutes, and then decreases; this is followed by a second prolonged phase of vasoconstriction which reaches a maximum after several hours and cannot be completely reversed after reexposure to normoxia, in contrast to the first acute phase [38, 39]. Von Euler and Liljestrand described this effect in 1946 in the cat and were the first to speculate that this mechanism may be responsible for ventilation–perfusion matching during regional hypoxia in the lung [40]. Indeed, regional alveolar hypoxia (e.g., due to alveolar hypoventilation) results in constriction of precapillary vessels at the entrance of the pulmonary acinus [41], which serves to redistribute blood from poorly ventilated to well ventilated alveoli and thus decreases pulmonary shunt flow, in order to optimize arterial oxygenation. Despite many years of research, the underlying mechanisms of HPV are not completely understood. The trigger for acute HPV seems to be located in the pulmonary arterial smooth muscle cells (PASMC), while initiation of sustained HPV may also depend on the presence of the endothelium [42] which is also a major modulator of HPV (e.g., via release of nitric oxide [NO]). Although the role of the endothelium is not completely resolved, it is commonly accepted that HPV is an adaptive physiologic mechanism attributed to the lung itself, because HPV is preserved in patients after lung transplantation [43].

Mitochondria or other oxygen-consuming organelles or enzymes in the PASMC, such as reduced nicotinamide adenine dinucleotide phosphate (NADPH) oxidases, may sense hypoxia and transfer the signal via alteration of the level of reactive oxygen species (ROS) or the cellular redox state to sarcoplasmic and plasmalemmal ion channels, such as potassium channels, transient receptor potential channels, and L-type calcium channels, which cause an intracellular calcium increase and vasoconstriction. Prolonged HPV may also be regulated by calcium sensitization via rho kinase and an alteration in the adenosine triphosphate (ATP)/adenosine monophosphate (AMP) ratio. However, the exact sequence of signal transduction and the primary oxygen sensor remain unknown (see reviews [56–59]). Particularly, the exact role of ROS as mediators in HPV, the effect of hypoxia on their levels, and the identities of their interaction partners (e.g., phospholipases and protein kinases) are not completely understood. Although chronic hypoxia-induced alterations of the pulmonary vasculature involve other pathologic mechanisms in addition to HPV, the identification of mechanisms regulating HPV has helped to uncover novel therapeutic approaches for PH (e.g., targeting the NO–cyclic guanosine monophosphate [cGMP] pathway with sildenafil) [60].

Moreover, HPV is of clinical significance, because decreased HPV (which can occur, for example, during anesthesia [61], pulmonary inflammation [e.g., sepsis [62]], or the hepatopulmonary syndrome [63]) can lead to arterial hypoxemia. By contrast, exaggerated global HPV can aggravate PH, and inhomogeneous HPV may contribute to the development of HAPE as described in a later section.

### 2.3. Acute Maladaptation

AMS is a combination of unspecific symptoms typically occurring in nonacclimatized individuals traveling above 2500 m. Its onset is within the first 6–12 hours after arrival at high altitude [6, 7, 12]. Typical symptoms are headache followed by at least one more of the following symptoms: loss of appetite, nausea, vomiting, dizziness, insomnia, and fatigue [6, 7]. Hitherto there are two hypotheses on the underlying pathogenesis.

(1) The fall in arterial oxygen concentration that accompanies an ascent to high altitude leads to an increase in the perfusion of the central nervous system (CNS) [64–66]. At the same time, autoregulation of the cerebral vessels is impaired [67, 68] and concentrations of circulating radicals [67, 69–71] and vascular endothelial growth factor (VEGF) [72] are increased. These circumstances may lead to increased permeability of the blood–brain barrier and thus result in extracellular edema. This hypothesis was supported by magnetic resonance imaging studies of healthy volunteers exposed to normobaric hypoxia [73, 74].

(2) Other data suggest that AMS is not associated with disruption of the blood–brain barrier [67]. Within the magnetic resonance imaging studies described above, asymptomatic individuals and those who developed AMS showed similar degrees of extracellular edema [73, 74], whereas those with AMS presented with an additional intracellular fluid accumulation, the quantity of which correlated with the degree of symptoms. In skeletal muscle cells, hypoxia-induced intracellular liquid accumulation has been shown to be related to impaired function of the Na^+^K^+^-ATPase [75]. The same impairment could be the cause of the previously mentioned intracellular fluid retention within the CNS [73]. Free circulating radicals are thought to reduce the activity of the Na^+^K^+^-ATPase, leading to an osmolarity-triggered fluid shift and thus swelling of astrocytes [73, 74]. The latter, via various mechanisms, is thought to lead to elevated NO synthesis [76] which, together with plasma membrane-destabilizing free radicals as well as VEGF, would result in irritation of the sensory trigeminal fibers, triggering the typical headache [77].

AMS itself is benign but severe cases may, if countermeasures are not undertaken or if further ascent is undertaken, result in HACE [7]. Typical signs of the latter are awareness alteration and/or ataxia. Without therapy, death may occur through cerebral herniation. On a pathophysiological basis, extravasation due to hyperperfusion is thought to contribute [6]. However, keeping the known pathomechanism of AMS in mind, cytotoxic edema will also be present in HACE [78]. The major contributor to the increased intracranial pressure is thought to be the extracellular fluid shift [79].

Exposure to hypoxia (normobaric or hypobaric) leads to inhomogeneous HPV, resulting in an uneven perfusion of the lung with nonperfused areas next to hyperperfused areas [80]. The high capillary pressure in the latter may result in exudation, indicating the beginning of HAPE [11, 81, 82]. Chest X-rays and computerized axial tomography scans conducted in the early stages of HAPE show patchy pulmonary infiltrate, sometimes with peripheral predominance. With progression of HAPE these areas grow and merge to produce a homogeneous distribution [83], and bronchoalveolar lavage fluid contains exudate rich in proteins as well as erythrocytes as a sign of mild hemorrhage [82, 84].

Typical symptoms of HAPE are breathlessness, initially dry cough that eventually becomes productive with sputum turning from white to pink, tachycardia, and sometimes cyanosis [1, 7]. These symptoms develop in susceptible individuals within the first 2–4 days after arrival at altitudes above 2500 m. The incidence depends on the ascent velocity and final altitude as well as individual susceptibility [7, 11]. Individuals at risk typically exhibit a marked rise in pulmonary arterial pressure (PAP) when exposed to hypoxia as a result of an exaggerated HPV [7], as well as a greater rise in PAP on exercise under normoxic conditions [7] and a reduced HVR compared with nonsusceptible controls [6, 85, 86].

### 2.4. Treatment of Acute Complications

AMS is typically a self-limiting condition that will resolve after 3-4 days [87]. However, if symptoms persist, descent to a lower altitude is recommended. Furthermore, supplemental oxygen (2–4 L/min) will reduce the symptoms within 15–30 min [87, 88]. Besides prevention through slow ascent and inclusion of resting days between ascents [89, 90], AMS can also be prevented effectively by acetazolamide or dexamethasone [91–93], and these two medications are each recommended by the Wilderness Medical Society with an evidence level of 1A [91–94]. However, acetazolamide has to be started the day before the ascent and is thus unsuitable for people who need to make unplanned, excessively rapid ascents, for instance, emergency rescue personnel [94, 95]. Dexamethasone should be considered first in such cases [96]. In 2014, Zheng et al. [97] showed that inhaled budesonide is able to prevent AMS to the same extent as oral dexamethasone [97]. As peak serum levels of budesonide are much lower than those of orally administered dexamethasone [98–100] the effect of budesonide is thought to be more focused on the lung tissue. The mechanisms underlying the effects of dexamethasone as well as inhaled budesonide are not fully understood, but dexamethasone is thought to increase the concentration of apical alveolar membrane Na^+^ channels as well as basal Na^+^K^+^-ATPase [101], stimulate surfactant secretion [102], and prevent protein exudation [103].

The first line of defence against HACE lies in its prevention through adequate acclimatization by slow ascent [90]. Dexamethasone is a possible treatment option but, in contrast to acetazolamide, dexamethasone does not facilitate acclimatization and thus may lead to a false sense of security. Its real potential lies in its possible stabilizing effect as a rescue drug for patients with HACE prior to descent [104, 105].

HAPE may be prevented by slow ascent and allowing adequate time to acclimatize, as mentioned above [7]. In addition, there are several medical options for the reduction of HAPE incidents: the phosphodiesterase type 5 (PDE5) inhibitor tadalafil (10 mg twice daily [BID]) and dexamethasone (8 mg BID) have been well evaluated [106] as has slow-release nifedipine (30 mg BID) [9]. When HAPE is evolving, the patient should descend to lower altitudes and receive immediate supplemental oxygen therapy (2–4 L/min) if possible [7, 107]. In terms of pharmacological therapy, adjunctive treatment with nifedipine is the current standard, while the use of PDE5 inhibitors requires further evaluation [94].

In cases of HACE and HAPE where descent is not possible, alternative options are supplemental oxygen and a portable inflatable hyperbaric chamber that is able to increase air pressure to the level found at an altitude of approximately 1500 m [108, 109].

## 3. Long-Term Changes of the Cardiopulmonary System due to Hypoxia

### 3.1. Hypoxia-Induced PH: Pathomechanisms and Preclinical Studies

Exposure to chronic hypoxia results in pulmonary vascular remodeling which is characterized by specific alterations of the large and small pulmonary vessels. These changes lead to PH and increased right ventricular (RV) afterload which can be further aggravated by increased blood viscosity [110]. Large pulmonary vessels show increased stiffening, while small pulmonary vessels exhibit thickening of the adventitial and medial layer and muscularization of formerly nonmuscularized precapillary vessels (“de novo” muscularization). Chronic hypoxia-induced vascular alterations are completely reversible after reexposure to normoxia.

**Figure 1:**
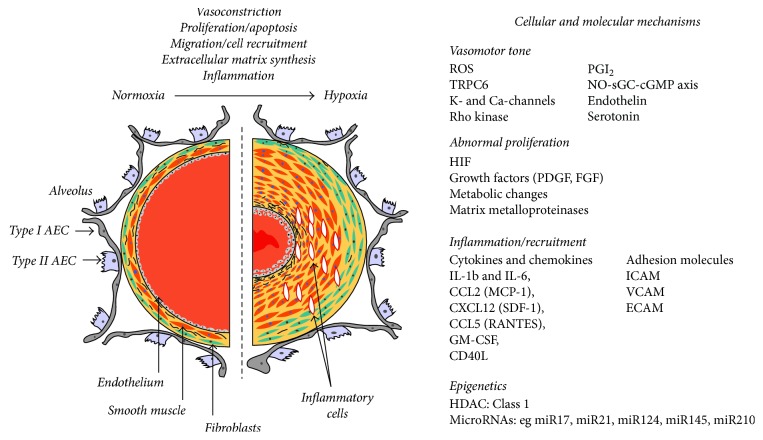
Mechanisms of vascular remodeling in chronic hypoxia (from [16], permission granted). AEC: alveolar epithelial cell; CCL: C-C motif chemokine ligand; CD40L: CD40 ligand; CXCL: C-X-C motif chemokine ligand; ECAM: endothelial cell adhesion molecule; FGF: fibroblast growth factor; HDAC: histone deacetylase; GM-CSF: granulocyte macrophage colony stimulating factor; HIF: hypoxia-inducible factor; ICAM: intercellular adhesion molecule; IL: interleukin; NO-sGC-cGMP: nitric oxide-soluble guanylate cyclase-cyclic GMP; MCP: monocyte chemoattractant protein; PDGF: platelet-derived growth factor; PGI_2_: prostacyclin; RANTES: regulated upon activation, normally T-expressed, and presumably secreted; ROS: reactive oxygen species; SDF: stromal cell-derived factor; TRPC6: transient receptor potential cation channel 6; VCAM: vascular cell adhesion molecule.

On a cellular level, the remodeling of the small vessels is caused by increased proliferation and migration and decreased apoptosis of PASMC (Figure 1) [16], and the affected cells seem to comprise a specific subpopulation of PASMC that are not well differentiated. Additional alterations include increased proliferation and migration of fibroblasts that have the ability to differentiate into smooth muscle cell-like cells and secrete matrix proteins. It is possible that pericytes and circulating bone marrow cells also contribute to the pathogenesis of vascular remodeling; however, species-specific differences exist [111, 112]. Compared with human forms of pulmonary arterial hypertension and other non-hypoxia-induced forms of PH, chronic hypoxia induces less alteration of the intima, specifically no formation of neointima and plexiform lesions [111]. However, alterations of endothelial cells also play a prominent role in chronic hypoxia-induced PH, as they release increased levels of vasoconstrictive, proproliferative factors (e.g., endothelin [ET] -1, angiotensin II, VEGF, and platelet-derived growth factor- [PDGF-] B) and reduced levels of vasodilatory, antiproliferative mediators (NO and prostaglandin I_2_), as well as increased amounts of adhesion molecules, cytokines, procoagulatory factors, and matrix proteins, which interact with adjacent cells and attract circulating immune and progenitor cell types [112, 113].

Hypoxia-induced endothelial stimuli and circulating systemic factors, as well as hypoxia per se, activate intracellular signaling cascades in PASMC involving tyrosine kinases, mitogen activated protein kinases, protein kinase C, phosphatidylinositol 3 kinase, SMAD phosphorylation, calcium inflow, and rho kinases, which all regulate cellular contractility, proliferation, and differentiation, as well as synthesis of matrix proteins. By contrast, antiproliferative signaling pathways which are regulated by cGMP or cyclic AMP become less active. Development of hypoxia-induced PH is further promoted by intrinsic genetic, epigenetic, and acquired factors, such as bone morphogenetic protein receptor-2 mutations and hormones [112].

Many of these factors have been targeted with investigational therapies in animal models of chronic hypoxia-induced PH, and beneficial effects have been shown. However, only a few targets have made the transition into clinical trials (for hypoxia-induced and/or other forms of PH), such as the PDGF pathway which was addressed by tyrosine kinase inhibitors [114, 115] and the NO–cGMP pathway which was addressed by activators/stimulators of the soluble guanylate cyclase [116, 117]. Further details of clinical studies in hypoxia-induced PH are provided in Section 3.3.

Mediators between the aforementioned intracellular signaling pathways and hypoxia, which may act as chronic hypoxic oxygen sensing mechanisms, include the activation of hypoxia-inducible factor (HIF) 1, inhibition of mitochondria, and alterations in the release of ROS. HIF1 regulates transcription of proteins such as erythropoietin, VEGF, and ET1, as well as proteins that regulate cellular glycolytic and mitochondrial metabolism [118]. In accordance with the prominent role of HIF in hypoxia-induced PH, mice with inducible PASMC-specific HIF1*α* knock-out showed decreased development of PH [119]. The inhibition of mitochondrial metabolism and increased glycolytic ATP production (the so-called “metabolic switch”) that has been observed in PH results in altered ROS release, antiapoptotic effects, activation of proliferative transcription factors, increased supply of components for protein synthesis, and altered cellular calcium homeostasis [120, 121]. Inhibition or reversal of mitochondrial alterations at several levels of interaction with the cellular signaling pathways could inhibit development of hypoxia-induced PH in mice and rats [121–124]. ROS can interact with a plethora of redox-sensitive proliferative and antiapoptotic pathways and their role in conditions of chronic hypoxia is as controversial as their role in acute hypoxia. In this regard, both an increase [59, 125] and a decrease of ROS have been shown to stabilize HIF [126]. Animal studies suggest that ROS scavenging may be beneficial in chronic hypoxia-induced PH under certain circumstances [127–129].

### 3.2. Long-Term Adaptations in High-Altitude Populations

**Figure 2:**
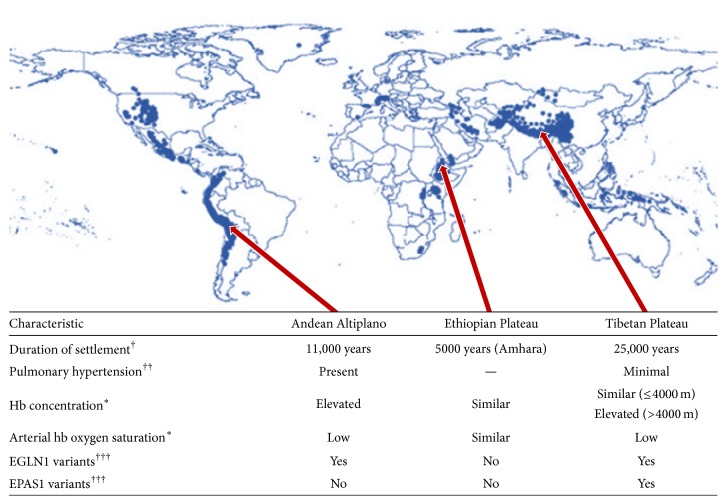
Map showing populated regions at altitudes of 2500 m or higher (from [17], permission granted), and characteristics of three major high-altitude populations. ^*∗*^Compared with sea-level populations at low altitude (see [18, 19]). EGLN1: hypoxia-inducible factor prolyl 4-hydroxylase 2; EPAS1: hypoxia-inducible factor-2*α*; hb: hemoglobin. †: [20, 21], ††: [22–24], †††: [25].

Studies of native high-altitude populations have also provided information regarding the mechanisms involved in (mal) adaptation to long-term hypobaric hypoxia. At varying times in history, humans colonized multiple high-altitude locales, including the Tibetan Plateau, the Andean Altiplano, and the Semien Plateau of Ethiopia [130]. The adaptation of these large populations to chronic hypoxia has been extensively studied (Figure 2). The Tibetan population has been a particular focus of research, because Tibetans are believed to have moved to the Tibetan Plateau (average elevation of 4000 m) almost 25,000 years ago, which would have given them more time to adapt to chronic hypoxia than other high-altitude human populations such as the native inhabitants of the Andean Altiplano (settled 11,000 years ago) and the Amhara population in Ethiopia (settled 5000 years ago [20]).

#### 3.2.1. Pulmonary Vascular System

Interestingly, high-altitude populations show relevant differences in their pulmonary vascular response to chronic hypoxia. Of note, Tibetans exhibit almost normal PAP, show minimal hypoxic PH [23], and lack the typical pathological findings of vascular remodeling [24]. By contrast, PH and CMS, including pathophysiological remodeling of the pulmonary arteries, are evident among Andeans [22]. These differences are most probably the result of the Tibetan population living above 3000 m for thousands of years longer than the Andean population.

In general, it is believed that increases in hematocrit and hemoglobin (hb) concentration negatively influence pulmonary pressures and resistance [131]. In this context, it is noteworthy that Tibetan highlanders have significantly lower hb concentrations than Andean highlanders or Han Chinese migrants to high altitude [132]. Highlanders undergoing right heart catheterization while being physically challenged were also found to have reduced pulmonary vascular distensibility with impaired and inadequate response of the CO to exercise [133, 134]. However, subsequent studies of highlanders showed a wide range of responses of the pulmonary vascular system to exercise [135, 136]. Overall, results indicate that pulmonary vascular distensibility and CO response are impaired in highlanders, with individual and ethnic variability.

Interestingly, manifest hypoxic PH in highlanders is almost reversible after two years of low-altitude exposure [137]. Substantial alterations in the release of vasodilatory factors, especially NO, are believed to contribute to the high-altitude phenotypes. Compared with lowland inhabitants, Tibetan highlanders exhibit higher levels of circulating NO [138], as well as a higher NO transfer rate within the pulmonary circulation (implying an enhanced pulmonary vasodilatation) [139].

#### 3.2.2. Genomic Studies

The local variability in the response to chronic hypoxia raised the question whether different genes might have been influenced by natural selection in the three major high-altitude populations. The HIF system has been described as the major genetic pathway [132], and HIF is believed to control hundreds of genes in response to cellular hypoxia.

In Tibetan highlanders, variants in several major HIF upstream genes such as EPAS1 (HIF-2*α*) and EGLN1 (HIF prolyl 4-hydroxylase 2) have been identified. It is believed that these variants contribute to the low hb concentrations in Tibetans [25, 140, 141]. In particular, a missense mutation in EGLN1 prevents hypoxia-induced and HIF-mediated enhancement of erythropoiesis [142]. Moreover, HMOX2 (heme oxygenase 2; downstream of HIF) has recently been identified as a relevant modifier of hb metabolism and contributor to high-altitude adaptation in Tibetan highlanders [143]. In addition, variants of the major upstream transcriptional regulator EPAS1 were also significantly associated with low hb concentrations in Tibetan highlanders [144]. The low hb concentration and concomitant reduction of blood viscosity compared with acclimatized lowlanders might be an important mechanism to maintain cardiopulmonary circulation in Tibetan highlanders [16].

Genome-wide studies of Andean highlanders showed an overlap with Tibetan highlanders for variation in EGLN1 but not EPAS1 [25]. Nevertheless, no relevant association between EGLN1 genotype and hb levels was evident in Andean highlanders. In addition, variants in EGLN1 and EPAS1 did not significantly contribute to Ethiopian hb concentrations [20]. However, high-altitude adaptation in Ethiopian highlanders is believed to be regulated via several different genes involved in vascular physiology such as CXC17 (CXC chemokine 17) and PAFAH1B3 (platelet activating factor acetylhydrolase 1b catalytic subunit 3) [145, 146]. Although the large highland populations have developed different genetic variants, some major pathways show a relevant overlap. Many other variants in major genetic pathways have been discovered, but these genetic variants alone might not be sufficient to explain fully the adaptation to chronic hypoxia. It is possible that other pathways will be discovered from studies in larger cohorts.

### 3.3. Clinical Studies

An overview of clinical studies of potential treatments for high altitude PH is presented in Table 2. The 2005 consensus statement on high altitude PH included results from two studies [8]. The effect of nifedipine was assessed in a case-control study that included 31 patients with high altitude PH diagnosed and treated in Bolivia at an altitude of 3600 m [44]. A 20% decrease in pulmonary arterial systolic pressure (PASP) was noted in two-thirds of the patients (classed as responders). In terms of limitations, it should be noted that the study was based on echocardiography only. In addition, although CO increased to a greater extent in responders than in nonresponders, heart rate and systemic systolic blood pressure did not show responses consistent with the pulmonary vascular response (heart rate increased to a similar extent in responders and nonresponders, and systemic systolic blood pressure showed a greater decrease in nonresponders than in responders) [44]. In another study in Bolivia (also at an altitude of 3600 m), the effect of isovolemic hemodilution was assessed in eight native residents [45]. Three of the eight participants had high altitude PH. Isovolemic hemodilution led to an increase in CO in all three participants with PH but had no consistent effect on mean PAP. Based on the data available at the time, the 2005 consensus statement recommended migration to low altitude as the ideal treatment for high altitude PH and noted that there was an urgent need for randomized controlled trials of possible alternatives such as calcium-channel blockers, ET receptor antagonists, prostaglandins, and PDE5 inhibitors [8].

In 2005, a randomized controlled trial of the PDE5 inhibitor sildenafil in 22 patients with high altitude PH in Kyrgyzstan was published [46]. The patients were randomly assigned to receive sildenafil 25 mg or 100 mg three times daily or placebo. Right heart catheterization was carried out at baseline and after 12 weeks of treatment. The patients receiving active treatment had a significantly greater reduction in PAP (−6 mm Hg) than those receiving placebo (+1 mm Hg). Changes in pulmonary vascular resistance and CO showed no difference between the sildenafil and placebo groups, but the higher dose of sildenafil increased CO and decreased pulmonary vascular resistance much more than the lower dose. The placebo-corrected increase in six-minute walking distance was more than 40 m for both sildenafil doses [46]. A 2010 meta-analysis of ten trials came to the conclusion that PDE5 inhibitors reduce PASP and have beneficial effects in patients with high altitude PH [47]. The NO−cGMP pathway has also been targeted by the soluble guanylate cyclase stimulator riociguat, which reduced PAP and pulmonary vascular resistance in volunteers during exercise at a simulated altitude of ~4600 m [48].

Measures of high altitude PH were followed as secondary endpoints in a study of acetazolamide in 55 patients with CMS in Peru [49]. After a double-blind, randomized, placebo-controlled phase, all patients received open-label acetazolamide. The increased hematocrit improved significantly with acetazolamide compared with placebo. No echocardiographic measures of high altitude PH improved with acetazolamide compared with placebo. However, CO improved significantly from baseline in the open-label phase (+1 L/min). The patients treated in this study most likely had no PH at baseline (echocardiographic PASP: 34 mm Hg plus right atrial pressure) [49].

The rho kinase inhibitor fasudil showed impressive effects in an acute hemodynamic study in 19 Kyrgyz patients living at altitudes above 3200 m with high altitude PH [50]. The placebo-controlled cross-over design allowed two acute echocardiographic evaluations of the right heart before and after infusion of fasudil (1 mg/min) or placebo over 30 min. Following fasudil infusion, PASP decreased by 10 mm Hg from a baseline of 52 mm Hg and CO increased by 0.5 L/min from a baseline of 6.1 L/min; neither parameter showed any change from baseline following placebo infusion [50].

Bosentan, an ET receptor antagonist approved for the treatment of pulmonary arterial hypertension, was tested as prophylaxis in healthy volunteers (maximal oxygen uptake: 52 mL/kg·min) taken by motor vehicle to an altitude of 3800 m for evaluation of echocardiographic parameters and exercise capacity [51]. Compared with placebo, bosentan was associated with a greater increase from sea-level baseline in PASP (+15 mm Hg [bosentan] versus +8 mm Hg [placebo]) and lower oxygen saturation during exercise (78% versus 85%). Other relevant measures did not differ [51]. However, an acute hemodynamic study showed a beneficial effect of bosentan on PASP (measured by echocardiography) in 15 Kyrgyz patients with invasively proven high altitude PH: a single oral dose of bosentan led to a decrease in PASP from 46 to 37 mm Hg after 3 h, while CO and pulsoxymetric saturation remained stable [52]. Another echocardiography study in healthy volunteers showed that a single dose of bosentan blunted the rise in PASP caused by acute (90 min) hypoxia exposure [53].

The effect of inhaled iloprost, a prostanoid therapy approved for the treatment of pulmonary arterial hypertension, was assessed in an echocardiography study of 12 healthy volunteers at an altitude of 5050 m. The results suggested that a prolonged stay at high altitude led to impairment of RV systolic and diastolic function which was not reversed by inhalation of a single dose of iloprost (5 *μ*g) [54].

The effect of iron depletion or supplementation on PASP was evaluated in two randomized, controlled, echocardiography studies, albeit not in patients with high altitude PH: one study assessed healthy volunteers ascending to 4340 m and the other study enrolled patients with CMS and PASP within the normal range (mean: 37 mm Hg) [55]. The estimated increase in PASP in healthy volunteers after ascent was partly reversed by iron supplementation (reduction by 6 mm Hg), but never reached pathological levels. In patients with CMS, progressive iron deficiency (induced by venesection and isovolemic donation of 2 L of blood) increased the estimated PASP by 9 mm Hg. Subsequent iron replacement did not change the PASP [55].

#### 3.3.1. (Possible) Treatment Options

As for other conditions at high altitude, descent is life saving for severe cases [16]. However, if descent is not possible, pharmacologic treatment can be considered. PDE5 inhibitors have the broadest evidence base to date, and high doses (e.g., 100 mg three times daily) of sildenafil could be considered. Further studies of pharmacologic treatments for high altitude PH are needed.

## 4. The Right Ventricle in Hypoxia

In hypoxic conditions such as in high altitude, right sided heart failure due to HPV and pulmonary vascular remodeling with the resulting increase in afterload is one of the most feared life-threatening diseases. Only 1% of previously healthy individuals with pulmonary vascular hyperreactivity to hypoxia and severe hypoxic PH are at risk of developing right heart failure in high altitude [131, 147]. The incidence of right heart failure in hypoxic conditions, for instance, at high altitude, is by no means proportional to high pulmonary pressure. It remains to be elucidated why some high-altitude residents with high PAP are able to live “normal” lives with no restrictions in their activities [148], whereas others develop right heart failure.

### 4.1. How Does the Right Ventricle React to Hypoxic Conditions?

The most illuminating studies in this context are those conducted at high altitude or in conditions similar to the hypoxic conditions of high altitude. Huez et al. [149] exposed 25 healthy volunteers to hypoxia for 90 min (fraction of inspired O_2_: 0.12) or dobutamine (titrated to produce the same effect on heart rate without changing pulmonary vascular tone) and explored the effects on RV and left ventricular function using standard Doppler echocardiography, pulsed tissue Doppler imaging, and longitudinal systolic strain and strain-rate imaging. Hypoxia and dobutamine both increased CO and correspondingly tricuspid regurgitation velocity as an index for PAP. Conventional echocardiography and tissue Doppler imaging revealed that dobutamine increased RV indices of systolic function (such as RV area shortening fraction, tricuspid annular plane systolic excursion [TAPSE], or systolic ejection wave velocity [*S*] at the tricuspid annulus), whereas hypoxia did not. Accordingly, longitudinal wall motion analysis revealed that* S*, systolic strain, and strain rate on the RV free wall and interventricular septum were increased by dobutamine but were not affected by hypoxia. These results indicate that systolic function of the right ventricle is not altered in hypoxia. Instead, early diastolic function appears to be affected; tissue Doppler imaging revealed that hypoxia increased the isovolumic relaxation time relative to the RR interval and delayed the onset of the* E* wave at the tricuspid annulus.

Echocardiographic measurements were performed by the same group [150] in 15 healthy lowlanders at different altitudes (sea level; <24 h after arrival in La Paz, Bolivia, at 3750 m; and after 10 days of acclimatization and ascent to Huayna Potosi, at 4850 m), and the results were compared with those obtained in 15 age- and body size-matched inhabitants of Oruro, Bolivia, at 4000 m. Acute exposure to high altitude in lowlanders caused an increase in mean PAP to 20–25 mm Hg, altered RV diastolic function (indicated by a decreased* E*/*A* ratio as well as a prolonged isovolumic relaxation time contributing to an increased RV Tei index), and maintained RV systolic function (measured by TAPSE and* S* at the tricuspid annulus). Compared with the lowlanders exposed to high altitude, the native highlanders had lower PAP but greater alteration in diastolic function, decreased TAPSE and* S* at the tricuspid annulus, and an increased RV Tei index. The cardiac adaptations to high altitude appeared qualitatively similar between both groups, but with significant deterioration of indices of systolic and diastolic function in high-altitude dwellers. According to the authors, these results could be an effect of a lesser degree of sympathetic nervous system activation in the native highlanders.

Allemann and colleagues [151] assessed the effects of rapid ascent to high altitude on PAP and right and left ventricular function by echocardiography in 118 nonacclimatized healthy children and adolescents. The echocardiography was performed at low altitude and 40 h after rapid ascent to 3450 m. PAP, estimated by measuring the systolic RV to right atrial pressure gradient, was significantly higher at high altitude than at low altitude. There was no depression in RV systolic function even in children with the most severe altitude-induced PH. RV systolic function even increased in the latter group. Naeije and colleague discussed another possible mechanism of right heart failure. Chronic hypoxia and relative hypercapnia could be a cause of salt and water retention in the mildly afterloaded right heart. This would lead to congestion, but remains to be proven in large studies [131].

Recently Crnkovic and colleagues [152] used a mouse model of chronic hypoxia to describe RV function in hypoxia. In this model, TAPSE was significantly decreased in hypoxia, whereas invasive measurements demonstrated a significantly increased maximal rate of rise in RV pressure (RV max dP/dt) and an unchanged RV contractility index. The authors concluded that RV systolic function is maintained in hypoxia, despite the decreased TAPSE. Due to its pressure-dependency, dP/dt [153] is a very vague parameter to describe RV contractility and therefore we need studies (in humans as well as animals) that investigate RV contractility and RV-arterial coupling using the accepted parameters Ees/Ea (ventricular end-systolic elastance/arterial elastance), in order to be able to measure the “correct” systolic function.

Recently Stembridge et al. [154] performed two-dimensional, Doppler, and speckle-tracking echocardiography on adolescent highland Sherpa (altitude: 3840 m; *n* = 26) compared with age-matched lowland Sherpa (altitude: 1400 m; *n* = 10) and lowland Caucasian controls (sea level; *n* = 30). The RV diastolic area showed no significant difference between the groups, whereas RV longitudinal strain and strain rate (reflecting contractile function) were lower in highland Sherpa compared with lowland Sherpa, with no difference between highland Sherpa and lowland Caucasian controls.

In conclusion, most of the studies investigating RV function in hypoxia are small and the majority of them used echocardiographic measurements to assess diastolic or systolic RV function. Further investigations should be done in order to assess the load-independent (as far as systolic function is concerned) parameter Ees and its relationship to Ea. All the other parameters mentioned above are indirect parameters, which at least give us some evidence that the systolic function of the right ventricle in most studies is not impaired, whereas the diastolic function is slightly reduced due to the increasing afterload. Investigations of Ees/Ea would enable us to measure directly the contractility of the right ventricle, providing us with more information about the reaction of the right ventricle to hypoxia.

## 5. Summary

Acute responses to hypobaric hypoxia include increased heart rate, increased erythrocyte concentration, increased breathing frequency and tidal volume (HVR), and HPV leading to increased pulmonary vascular resistance and right ventricular afterload. Acute maladaptation results in increased CNS perfusion and intracellular fluid accumulation (AMS, which can lead to potentially fatal HACE), as well as inhomogeneous HPV leading to hyperperfusion of some areas of the lung with subsequent exudation (HAPE). Acute maladaptation may be prevented by slow ascent and/or by administration of acetazolamide or dexamethasone; tadalafil and nifedipine are also options for reduction of HAPE events. Treatment of acute maladaptation involves descent to a lower altitude and/or administration of supplemental oxygen; dexamethasone may be used to stabilize patients with HACE prior to descent, and nifedipine is used as adjunctive therapy in HAPE.

Chronic hypoxia leads to pulmonary vascular remodeling (and thus PH) via HIF1 activation, mitochondrial inhibition, altered ROS release, and downstream alteration of many signaling pathways in endothelial cells and PASMC. The resulting increase in RV afterload can lead to the development of life-threatening right heart failure. Despite extensive study of physiological adaptations in native high-altitude populations and the evaluation of multiple potential pharmacological targets in animal models, few pharmacological treatments for hypoxia-induced PH have entered clinical trials. PDE5 inhibitors have the most evidence to date, and further studies of pharmacological therapies are needed. Data on RV function in hypoxia are also limited, and future studies should assess RV contractility directly by investigating Ees and Ea.

## Figures and Tables

**Table 1:** Categorization of altitude.

Altitude category	Height above sea level
(I) Moderate altitude	1500–2500 m
(II) High altitude	2500–3500 m
(III) Very high altitude	3500–5800 m
(IV) Extremely high altitude	>5800 m

**Table 2:** Clinical studies of potential treatments for high altitude PH.

Trial [reference]	Design	Study population (*n*)	Location (altitude)	Treatments	Main hemodynamic results
Antezana et al. 1998 [44]	Uncontrolled, open-label trial with case-control analysis (high versus low baseline Hb and PASP; responders versus nonresponders)	Native residents at high altitude (*n* = 31 [14 with PH])	La Paz, Bolivia (3500–4100 m)	Nifedipine 10 mg (1–3 doses at 30 min intervals; sublingual)	Two-thirds of participants overall showed response to nifedipine (>20% decrease in PASP), but systemic systolic blood pressure showed greater decrease in nonresponders than responders
Manier et al. 1988 [45]	Uncontrolled, open-label trial	Native residents at high altitude (*n* = 8 [3 with PH])	La Paz, Bolivia (3600–4200 m)	Isovolemic hemodilution	Isovolemic hemodilution led to an increase from baseline in CO but had no consistent effect on mean PAP in participants with high altitude PH
Aldashev et al. 2005 [46]	Double-blind, randomized, placebo-controlled trial	Patients with high altitude PH (*n* = 22)	Naryn region, Kyrgyzstan (2500–4000 m)	Sildenafil 25 or 100 mg or placebo every 8 h for 12 weeks (tablets)	Sildenafil had a significant treatment effect versus placebo in terms of mean PAP (−6.7 mm Hg [95% CI: −11.6 to −1.8]; *p* = 0.010) and 6MWD (+43.5 m [95% CI 13.4 to 72.6]; *p* = 0.007)
Jin et al. 2010 [47]	Meta-analysis of randomized, controlled trials	Patients with high altitude PH (*n* = 218 [in 10 trials])	(>2500–5400 m)	PDE5 inhibitors	PDE5 inhibitors had a significant treatment effect versus control in terms of PASP at rest (weighted mean difference −7.5 mm Hg [95% CI: −10.9 to −4.2]; *p* < 0.0001), and no significant effect on systolic blood pressure and heart rate at rest and during exercise
Andrews et al. 2016 [48]	Open-label trial (hemodynamics evaluated during incremental exercise tests before and after administration of study drug)	Volunteers (*n* not reported)	Simulated altitude of ~4600 m	Riociguat 1 mg (single oral dose)	Riociguat led to a decrease in PAP and PVR at all levels of exercise intensity
Richalet et al. 2008 [49]	Double-blind, randomized, placebo-controlled trial, followed by an open-label trial after a 4-week washout period	Patients with CMS (*n* = 55)	Cerro de Pasco, Peru (4300 m)	Randomized phase: acetazolamide 250 mg or placebo daily for 12 weeks (oral) Open-label phase: acetazolamide 250 mg daily for 12 weeks (oral)	Randomized phase: acetazolamide had no significant effect on echocardiographic measures of high altitude PH compared with placebo Open-label phase: acetazolamide led to significant improvements from baseline in CO (original placebo and acetazolamide groups both +1 L/min [*p* < 0.001]) and PVR (original placebo group: −0.12 WU [*p* < 0.02]; original acetazolamide group: −0.19 WU [*p* < 0.001])
Kojonazarov et al. 2012a [50]	Double-blind, randomized, placebo-controlled, crossover trial	Patients with high altitude PH (*n* = 19)	Tien-Shan Mountains, Kyrgyzstan (3200–3600 m)	Fasudil hydrochloride hydrate 30 mg or placebo (IV infusion)	Fasudil infusion led to improvements from baseline in PASP (−10 mm Hg) and CO (+0.5 L/min), whereas placebo infusion did not (*p* < 0.001 for fasudil versus placebo)
Seheult et al. 2009 [51]	Double-blind, randomized, placebo-controlled, crossover trial	Nonacclimatized volunteers (*n* = 8)	White Mountains, CA, USA (3800 m)	Bosentan 125 mg or placebo twice daily for 5 days before ascent and 2 days at high altitude (oral)	After ascent to high altitude, PASP increased from sea-level baseline to a greater extent with bosentan (+15 mm Hg) than with placebo (+8 mm Hg)
Kojonazarov et al. 2012b [52]	Uncontrolled, open-label trial	Patients with high altitude PH (*n* = 15)	Tien-Shan Mountains, Kyrgyzstan (2500–3800 m)	Bosentan 125 mg (single oral dose)	Bosentan led to a decrease in PASP from 46 to 37 mm Hg after 3 h, while CO remained stable
Pham et al. 2012 [53]	Double-blind, randomized, placebo-controlled, crossover trial	Volunteers (*n* = 15)	Acute (90 min) normobaric hypoxia equivalent to altitude of ~4300 m	Bosentan 250 mg or placebo (single oral dose)	Compared with placebo, bosentan blunted the hypoxia-induced rise in PASP by 6.4 mm Hg (*p* = 0.063) and 5.2 mm Hg (*p* = 0.002) in participants with and without a history of high altitude pulmonary edema, respectively
Kortekaas et al. 2009 [54]	Double-blind, randomized, placebo-controlled, crossover trial	Volunteers (*n* = 12)	Dhaulagiri, Nepal (5050 m)	Iloprost 5 *µ*g or placebo (single inhaled dose) at sea level and after 14-day trek to high altitude	TAPSE and tricuspid inflow peak velocities were decreased after trekking from sea level to high altitude, suggesting impaired right ventricular systolic and diastolic dysfunction; a single dose of inhaled iloprost did not reverse these changes
Smith et al. 2009 [55]	Two double-blind, randomized, placebo-controlled trials, one in healthy volunteers and one in patients with CMS (the latter also had a crossover phase)	Native sea level volunteers (*n* = 22) Native high altitude residents with CMS (*n* = 11)	Cerro de Pasco, Peru (4340 m)	Sea level volunteers: Fe(III)-hydroxide sucrose 200 mg or placebo (IV infusion) on third day after ascent to high altitude by road Patients with CMS: isovolemic hemodilution followed by Fe(III)-hydroxide sucrose 400 mg or placebo (IV infusion)	Sea level volunteers: at high altitude, iron infusion reduced PASP by 6 mm Hg (95% CI: 4 to 8; *p* = 0.01) Patients with CMS: iron depletion by hemodilution increased PASP from baseline by 9 mm Hg (95% CI: 4 to 14 mm Hg; *p* = 0.003); subsequent iron replacement had no acute effect on PASP

6MWD: 6-minute walking distance; CI: confidence interval; CMS: chronic mountain sickness; CO: cardiac output; Hb: hemoglobin; IV: intravenous; PAP: pulmonary arterial pressure; PASP: pulmonary arterial systolic pressure; PDE: phosphodiesterase; PH: pulmonary hypertension; PVR: pulmonary vascular resistance; TAPSE: tricuspid annular plane systolic excursion.
